# Effects of ischemic postconditioning on expressions of pentraxin-related protein 3 and neutrophil CD11b in the plasma of patients with acute myocardial infarction after percutaneous coronary intervention

**DOI:** 10.12669/pjms.322.9457

**Published:** 2016

**Authors:** Sheng-Hui Liu, Yu-E Huo, Xin-Wei Jia, Ya Li

**Affiliations:** 1 Sheng-Hui Liu, Department of Cardiology, Affiliated Hospital of Hebei University, Baoding 071000, China; 2 Yu-E Huo, Department of Cardiology, Affiliated Hospital of Hebei University, Baoding 071000, China; 3 Xin-Wei Jia, Department of Cardiology, Affiliated Hospital of Hebei University, Baoding 071000, China; 4 Ya Li, Department of Cardiology, Affiliated Hospital of Hebei University, Baoding 071000, China

**Keywords:** Myocardial infarction, Ischemic postconditioning, Neutrophil, Cell adhesion molecule, Pentraxin-related protein 3

## Abstract

**Objective:**

To evaluate the effects of ischemic postconditioning on expressions of pentraxin-related protein 3 (PTX3) and neutrophil CD11b in the plasma of patients with acute myocardial infarction (AMI) after percutaneous coronary intervention (PCI).

**Methods:**

Fifty-six patients who had AMI with ST-segment elevation were randomly divided into a control group and an ischemic postconditioning group (n=28). Both groups received emergency PCI. After recanalization of infarct-related arteries, the control group did not receive intervention within three minutes, while the ischemic postconditioning group was treated by low-pressure filling and emptying of balloon within one minute. The plasma expressions of PTX3 before and 24 hour after PCI were detected by ELISA, and those of neutrophil CD11b were detected by flow cytometry.

**Results:**

PTX3 and neutrophil CD11b expressions of the two groups were similar before PCI, but those of the ischemic postconditioning group significantly decreased 24 hour after PCI (P<0.05).

**Conclusion:**

Ischemic postconditioning lowered the expressions of PTX3 and neutrophil CD11b in AMI patients after PCI, inhibited inflammatory response, reduced the adhesion between leukocytes and endothelial cells, and protected the ischemic-reperfused myocardium.

## INTRODUCTION

Ischemic postconditioning, which refers to several cycles of reperfusion-ischemia performed immediately after reperfusion, can protect the myocardium by decreasing infarct area and relieving reperfusion-induced arrhythmias.^1^-^3^ On the other hand, inflammatory response plays an important role in the onset and progression of AMI. Whether ischemic postconditioning affects pentraxin-related protein 3 (PTX3) and CD11b as crucial inflammatory mediators has never been reported. Therefore, we aimed to assess the influence of ischemic postconditioning on PTX3 and CD11b expressions.

## METHODS

### Sampling and grouping

Fifty-six patients who had AMI with ST-segment elevation and treated in our hospital from June 2012 to December 2013 were selected, comprising 39 males and 17 females aged 36-73 years old. This study was approved by the ethics committee of our hospital, and written consent has been obtained from all patients. The time from onset to emergency coronary angiography (CAG) was less than 12 hour. The patients complicated with old myocardial infarction, infections, systemic autoimmune disease, connective tissue diseases, tumors or liver and kidney dysfunction were excluded.

The patients were randomly divided into a control group and an ischemic postconditioning group (n=28), and both subjected to routine CAG and percutaneous coronary intervention (PCI). In the Ischemic post conditioning group, Infarct-related artery (IRA) was recanalized, blocked by primary balloon angioplasty (PTCA) for 30 s within one minute. after reperfusion, reperfused for another 30 seconds after emptying of the balloon. This procedure was repeated three times, after which a stent was inserted. Control group: IRA was recanalized, without any intervention within three minutes after reperfusion. Afterwards, routine operations were performed. The baseline clinical data of the two groups were similar (P>0.05) (Table-I).

**Table-I:** Baseline clinical data.

Clinical characteristics	Control group (n=28)	Ischemic postconditioning group (n=28)
Age (year)	60.7±10.8	57.9±11.3
Male (case)	18 (64%)	21 (75%)
Hypertension (case)	16 (57%)	18 (64%)
Diabetic mellitus (case)	6 (21.4%)	5 (17.8%)
Hyperlipidemia (case)	13 (46.4%)	15 (53.6%)
History of smoking (case)	12 (42.9%)	14 (50%)
Infarct site		
Anterior wall (case)	20 (71.4%)	22 (78.6%)
Posterior wall (case)	8 (28.6%)	6 (21.4%)
Time from onset to puncture (h)	4.5±2.4	5.0±2.6
IRA		
Anterior descending artery (case)	20 (71.4%)	22 (78.6%)
Right coronary artery (case)	6 (21.4%)	5 (17.9%)
Circumflex artery (case)	2 (7.2%)	1 (3.5%)

### Detection indices

Venous blood (3 ml) was collected before and 24 hour after PCI. Plasma PTX3 concentration was detected by ELISA, with the sensitivity of <0.15 μg/L. The reagents were purchased from ALEXIS (USA). Anticoagulated blood (100 μL) was added CD11b-PE antibody, reacted at room temperature in dark for 20 minutes, added 2 ml of lysis buffer to eliminate erythrocytes, and washed twice with phosphate buffer. Neutrophil CD11b expression was detected by flow cytometry (BD, USA). Fluorescent signals were obtained in logarithmic form to calculate the average fluorescent intensity. Creatine kinase isoenzyme (CK-MB) level was measured every four hour before and after PCI until maximum.

### Statistical analysis

All data were analyzed by SPSS 11.5. The categorical data were expressed as mean ± standard deviation, and inter-group comparisons were performed with t test. The numerical data were compared by χ^2^ test. P<0.05 was considered statistically significant, and P<0.01 was considered extremely statistically significant.

## RESULTS

### Maximum CK-MB levels

The ischemic postconditioning group had significantly lower maximum CK-MB level than that of the control group (116.5±31.49 vs 144.6±41.3 U/L) (P<0.05).

### PTX3 and neutrophil CD11b expression levels

PTX3 and neutrophil CD11b expressions of the two groups were similar before PCI (P>0.05), but those of the ischemic postconditioning group significantly decreased 24 h after PCI (P<0.05) (Table-II).

**Table-II:** PTX3 and neutrophil CD11b expression levels (mean±SD).

Group	Before PCI (CD11b)	24 h after PCI (CD11b)	Before PCI (PTX-3)	24 h after PCI (PTX-3)
Control group (n=28)	405.62±61.63	498.71±83.78	6.06±3.17	7.36±4.25
Ischemic postconditioning group (n=28)	418.86±52.37	463.42±73.87^a^	6.13±3.69	6.58±4.18^a^

a P<0.05 vs. control group.

## DISCUSSION

At the beginning of reperfusion, repeated, transient recanalization and reclosure of the coronary artery can alleviate ischemia-reperfusion injury and protect the myocardium, also known as ischemic postconditioning. By establishing a canine model of ischemia-reperfusion, Zhao et al.^1^ found that ischemic postconditioning protected the myocardium from reperfusion injury, which has been verified in many other animal models. In 2005, Staat et al.^3^ first proved that ischemic postconditioning exerted the same effect in clinical practice. Subsequently, it has been reported that ischemic postconditioning exerted long-term improving effects on the myocardial perfusion of AMI patients after PCI, and their cardiac function.^4^-^7^ Ischemic postconditioning protects the heart by 1) improving hemodynamic indices (e.g. inhibiting the effects of myocardial infarction on blood pressure and heart rate), 2) decreasing infarct area, 3) reducing the release of myocardial enzymes (e.g. CK-MB and cTnl that mainly mark myocardial injury) and 4) mitigating free radical-induced myocardial injury.

As a member of the B2 integrin subfamily, CD11b mainly exists in neutrophils and monocytes, interacts with many types of ligands, and plays crucial roles in intercellular adhesion and inflammatory response. Under physiological conditions, it is only lowly expressed on the cell surface, which, however, increases obviously upon diseases.^8^ Since AMI is commonly accompanied by inflammatory response, neutrophil CD11b expression is bound to increase,^9^, ^10^ which then mediates the migration of numerous neutrophils to tissues around the related blood vessels by interacting with cell adhesion molecule ICAM-1 expressed in ligand-endothelial cells. As a result, cardiomyocytes are injured due to released oxygen radicals, platelet activating factor, collagenase, elastase, cathepsin, proteolytic enzymes and lipid metabolites.^11^

PTX3, as a newly discovered acute-phase protein upon inflammation, belongs to the hs-CRP superfamily. Michael et al.^12^ reported that PTX3 was highly expressed in AMI patients. By using immunohistochemical assays, Muller et al.^13^ found that pro-inflammatory cytokines such as LPS, IL-1b and TNF-α induced main pro-atherosclerotic cells (i.e. endothelial cells and monocytes) and cardiomyocytes to express PTX3, being closely associated with the onset and progression of inflammatory heart diseases. Inoue et al.^14^ reported that the expression level of PTX3 at the edge of atherosclerotic plaques exceeded that in the center, suggesting that activation of these plaques was positively correlated with PTX3 level. Latini et al.^15^ demonstrated that serum PTX3 could predict the 3-month mortality rate of AMI patients with ST-segment elevation, as a risk stratification predictor for death after myocardial infarction. Moreover, plasma PTX3 levels of patients with unstable angina and AMI patients with ST-segment elevation were measured within 24 h after chest pain, showing that PTX3 was positively correlated with the probability of cardiovascular events within 6 months and able to reflect local vascular inflammation and damage of the cardiovascular system.^16^

In summary, ischemic postconditioning decreased the expressions of PTX3 and neutrophil CD11b in AMI patients after PCI, and suppressed inflammatory response, thus relieving myocardial injury finally.
